# A ‘Landscape physiology’ approach for assessing bee health highlights the benefits of floral landscape enrichment and semi-natural habitats

**DOI:** 10.1038/srep40568

**Published:** 2017-01-13

**Authors:** Cédric Alaux, Fabrice Allier, Axel Decourtye, Jean-François Odoux, Thierry Tamic, Mélanie Chabirand, Estelle Delestra, Florent Decugis, Yves Le Conte, Mickaël Henry

**Affiliations:** 1 INRA, UR406 Abeilles et Environnement, Domaine Saint-Paul, CS 40509, 84914 Avignon, France; 2 UMT PrADE, CS 40509, 84914 Avignon, France; 3 ITSAP-Institut de l’Abeille, Domaine Saint-Paul, CS 40509, 84914 Avignon, France; 4 ACTA, CS 40509, 84914 Avignon, France; 5 INRA, UE1255 Entomologie, 17700 Surgères, France; 6 ADAPIC, Cité de l’Agriculture, 45921 Orléans, France

## Abstract

Understanding how anthropogenic landscape alteration affects populations of ecologically- and economically-important insect pollinators has never been more pressing. In this context, the assessment of landscape quality typically relies on spatial distribution studies, but, whether habitat-restoration techniques actually improve the health of targeted pollinator populations remains obscure. This gap could be filled by a comprehensive understanding of how gradients of landscape quality influence pollinator physiology. We therefore used this approach for honey bees (*Apis mellifera*) to test whether landscape patterns can shape bee health. We focused on the pre-wintering period since abnormally high winter colony losses have often been observed. By exposing colonies to different landscapes, enriched in melliferous catch crops and surrounded by semi-natural habitats, we found that bee physiology (i.e. fat body mass and level of vitellogenin) was significantly improved by the presence of flowering catch crops. Catch crop presence was associated with a significant increase in pollen diet diversity. The influence of semi-natural habitats on bee health was even stronger. Vitellogenin level was in turn significantly linked to higher overwintering survival. Therefore, our experimental study, combining landscape ecology and bee physiology, offers an exciting proof-of-concept for directly identifying stressful or suitable landscapes and promoting efficient pollinator conservation.

Anthropogenic effects on landscape (habitat loss, fragmentation and degradation) expose most insect pollinators to new and enduring environmental challenges and are primary drivers of their decline^1^,^2^,^3^,^4^,^5^. This represents a major conservation issue because insect pollination is vitally important to the maintenance of biodiversity and crop production^6^,^7^. Therefore, there is an urgent need to understand how landscape alteration affects those populations, and to promote landscape restoration, notably via agri-environment schemes (incentives for farmer to benefit the environment)^8^,^9^,^10^,^11^,^12^,^13^.

Traditionally, studies have focused on the relation between species distribution (e.g. presence/absence, abundance) and landscape patterns^1^,^3^,^6^,^14^,^15^,^16^,^17^,^18^,^19^. However, while informative, the assessment of disturbances is limited because the health state of the population is not considered and the deleterious effects of landscape alteration can only be detected once the population has started to decline^20^,^21^. A more powerful approach would be to characterize the specific mechanisms underlying the population response by combining physiological and ecological knowledge^21^. Indeed, the persistence of a population can be inferred by the health conditions of individuals within the population, and their physiological responses to environmental changes can provide an early indication of a stressful landscape^20^,^22^. But most importantly it provides a cause-and-effect relationship between landscape quality and population response, which has the potential to directly contribute to decision-making and support conservation policy^20^,^21^,^22^.

Floral resource availability in different landscape contexts has been linked to colony growth or productivity and variation in nutritional variables in both bumble bees^23^,^24^,^25^ and honey bees^26^,^27^,^28^,^29^,^30^. However, knowledge on the connection between landscape quality, notably landscape enrichment with floral resources, and bee health, is clearly limited. We therefore used a ‘Landscape physiology’ approach, integrating physiological data with landscape ecology^20^, to test *i*) the connection between bee health and landscape quality, and *ii*) whether agri-environment schemes can provide benefits to bee health. For that purpose, we exposed honey bee colonies (*Apis mellifera*) to different agricultural landscapes, either enriched or not by melliferous catch crops (environmentally friendly practices to promote bee forage) and surrounded by semi-natural habitats. We then assessed the link between the landscape quality (catch crop and semi-natural habitats), bee physiology and the consequential colony survival.

The study was performed during the pre-wintering period because severe winter mortality recently observed in honey bee colonies^31^,^32^ suggests that preparation for overwintering is especially challenging. Indeed, sufficient energetic reserves must be stored at the individual and colony level for a successful overwintering^33^. Bee health was assessed by determining fat body mass and the gene expression level of vitellogenin^28^,^34^. Both are physiological features of winter bees that arise during the autumn in temperate regions as an adaptation for surviving throughout the winter period. Indeed, winter bees have greater nutrient storage in the fat body and tolerance to oxidative stress than summer bees, due to the storage protein vitellogenin^35^,^36^. This ubiquitous protein, produced in the fat body, acts as an antioxidant and promotes the longevity of bees^37^. Its level is high in young bees but exhibits a negligible decline over time in winter bees as compared to summer bees^38^, likely explaining why winter bees are long-lived (several months) as compared to summer bees (4–6 weeks). Since fat body growth and vitellogenin production are both triggered by pollen intake^39^,^40^, we hypothesized a connection between their levels and the landscape-wide floral resource availability. In addition, we assessed the infestation levels of *Varroa destructor* as this parasitic mite is known to have detrimental effects on overwintering survival^35^.

## Results and Discussion

### Ecophysiological basis of bee health

Before wintering, colonies were set up either inside (n = 184 colonies split into 10 apiaries) or outside a melliferous catch crop area (n = 166 colonies split into 8 apiaries), following a control-*vs*-treatment experimental design (supplementary Figs S1 and S2). The paired control-*vs*-treatment experimental set-up was designed to avoid concomitant variations of semi-natural habitat land cover while varying catch crop treatment. Catch crop and semi-natural land cover values were therefore maintained uncorrelated (n = 18, Pearson’s correlation r = −0.17, P = 0.49).

We first explored the dataset for possible confounding effects due to year (winters 2012–13 and 2013–14) or colony allocation between treatments. No inter-annual variation in overwintering survival was detected when considering year effect alone (generalized linear mixed models (GLMM), df = 347, z = 1.19, P = 0.23) nor in combination with the fully parameterized survival model including physiological, brood and landscape covariates (df = 165, z = −0.426, P = 0.67). We could therefore consider the apiaries from different years as independent replicates within each beekeeping set-up. Furthermore, the random allocation of colonies led to apiaries with brood initial state independent from their landscape context, either considering colonies as independent entities (simple linear model, df = 345, catch crop land cover: t = 0.88, P = 0.38; semi-natural habitat land cover: t = 1.17, P = 0.24), or specifying a random grouping structure to properly account for the non-independencies within beekeeping set-ups (GLMM, df = 343, catch crop land cover: t = −1.03, P = 0.30; semi-natural habitat land cover: t = 1.04, P = 0.30).

**Figure 1:**
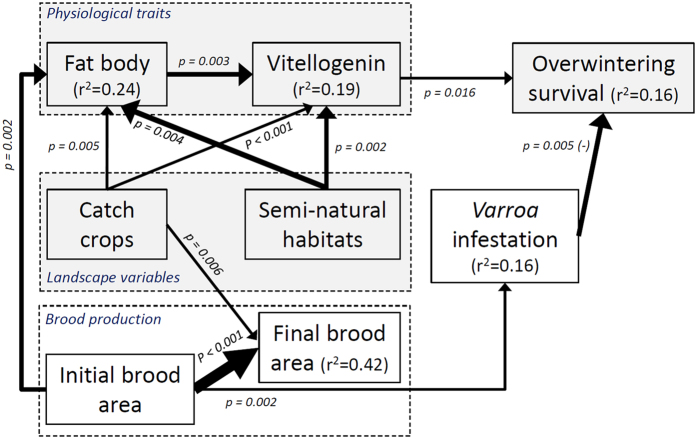
Path model revealing the ecophysiological basis of honey bee colony overwintering survival. Significance level is indicated next to each link. All links stand for positive effects, except *Varroa* infestation level that negatively affects overwinter survival. When at least two links reach the same box, their thickness is proportional to their effect coefficient (standardized on their respective range for direct comparison). Total explained variance (r^2^) is indicated in the box for each response variable in the causal chain. Landscape influence on bee health is highlighted in grey.

We then performed a path analysis to disentangle the direct and indirect dependencies of bee physiological traits and overwintering survival (n = 350 colonies) on landscape quality (gradients of catch crop from 0 to 0.315 km^2^ and semi-natural habitats from 0.04 to 2.652 km^2^), brood area and *Varroa* infestation level. Path analysis helps reconstruct the most plausible chain of causal links in multivariate datasets by assessing conditional independences among indirectly linked variables^41^,^42^. We identified the simplest path that did not deviate from conditional independence expectations while including only significant links (path analysis *d*-separation test, Fisher *C* = 30.08, df = 28, P = 0.36). This path model, that best captured the ecophysiological causal links behind overwintering survival, involved all studied variables (Fig. 1 and Table 1).

According to the path analysis, the initial colony level of brood had a direct and positive influence on *Varroa* infestation level and fat body mass of bees (GLMM with Gaussian distribution, df = 334, t = 3.16, p = 0.0017 and df = 169, t = 3.13, p = 0.0021, respectively; Fig. 1, Table 1 and supplementary Fig. S3). This could be easily explained by the fact that *i) Varroa* mites reproduce in brood cells, and *ii*) brood requires feeding by nurse bees, who take up nutrients from the fat body for secreting brood food via hypopharyngeal glands^43^. In addition, brood production was strongly determined by the initial colony level of brood (df = 341, t = 12.67, p < 0.001; supplementary Fig. S4) but did not affect bee physiological traits.

**Figure 2:**
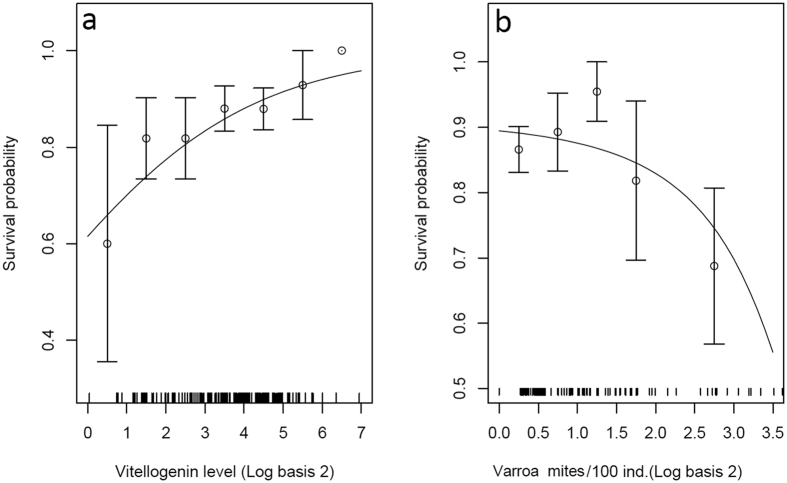
Representation of the path model links showing the influence of *Varroa* infestation and vitellogenin levels on overwintering survival. Survival probability was influenced positively by vitellogenin level (**a**) and negatively by *Varroa* infestation level (**b**). The continuous lines show model predictions. For the binary response variable (colony overwintering survival), data are represented as mean ± SE after being pooled into groups of consistent sizes. Tick marks show the position of raw data along the horizontal axis.

**Figure 3:**
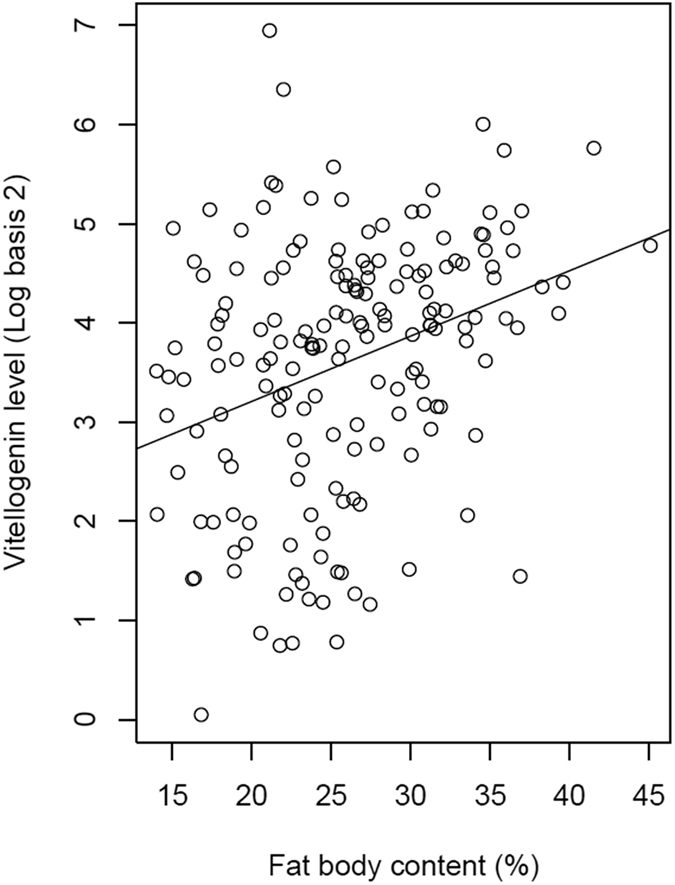
Relationship between fat body content and vitellogenin levels.

Regarding bee health, our data showed that overwintering survival was positively influenced by vitellogenin level (GLMM with binomial family distribution, df = 170, z = 2.41, P = 0.016; Figs 1 and 2a), as previously evidenced in different environmental contexts^28^,^34^. This suggests that this phospholipoglycoprotein involved in survival traits such as oxidative stress resilience, cellular immunity, and longevity^37^ can be employed as a predictive biomarker for monitoring honey bee populations. As expected, higher vitellogenin level was itself linked to higher fat body contents (df = 169, t = 2.94, P = 0.003; Figs 1 and 3) and overwintering was negatively influenced by *Varroa* infestation level during the pre-winter period (df = 170, z = −2.78, P = 0.005; Figs 1 and 2b). However, we did not find a link between vitellogenin and *Varroa* levels (t = −0.67, P = 0.50), contrary to a previous study, which showed that colonies that were not treated against *Varroa* (high mite infestation rate) had lower levels of vitellogenin in the fall compared to treated colonies^34^. One probable explanation is that our colonies were all treated against the mite by beekeepers and thus exhibited rather low *Varroa* infestation rates and negligible effects on vitellogenin levels^44^.

**Figure 4:**
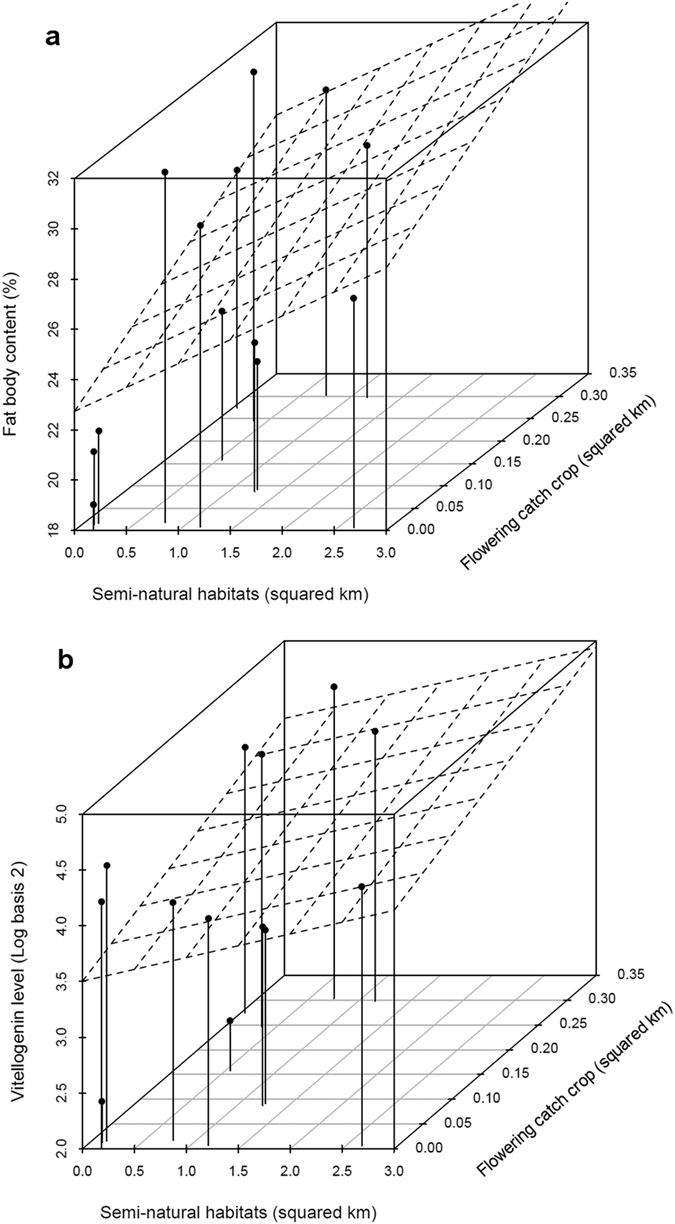
Representation of the path model links showing the influence of landscape quality variables on bee physiological traits. Melliferous catch crop and semi-natural habitats positively influenced the fat body (**a**) and vitellogenin levels (**b**). Fat body and vitellogenin values were averaged per apiary and plotted as a function of landscape metrics. Trends are depicted by the regression planes. A slight horizontal jitter was applied to separate overlying apiary data with equal flowering catch crop treatments.

Brood production, which was promoted by the presence of catch crops but not semi-natural habitats (t = 2.75, p = 0.0063 and t = 1.83, p = 0.068, respectively, Table 1 and supplementary Fig. S4), did not contribute to a better overwintering survival (df = 170, z = 2.32, p = 0.98). However, we found that landscape quality, whether melliferous catch crop or semi-natural habitat land covers, further facilitated the above-mentioned physiological causal chain, with a 1.6 and 1.9 times greater effect on vitellogenin and fat body, respectively, of semi-natural habitats as compared to catch crop (catch crop: fat body: df = 169, t = 2.84, P = 0.005; vitellogenin: t = 3.49, P < 0.001; semi-natural habitats: fat body: df = 169, t = 2.95, P = 0.003; vitellogenin: t = 3.10, P = 0.002; see Figs 1 and 4, and standardized estimates in Table 1 for effect size comparison). We therefore showed that bee health is affected by landscape patterns. More specifically, landscape enrichment with catch crops and semi-natural habitats promoted the physiological development of winter bees, which then enhanced the probability of overwintering survival. A similar result has been found in the United States when comparing the winter survival of colonies exposed to high or low potential forage^28^,^29^,^30^.

We could reconstruct a consistent ecophysiological causal chain behind overwintering survival, but there was still substantial variability inherent in the studied system (e.g. variability in bee physiological traits and brood production). In addition, many factors (e.g. pathogens, genotype and weather) can cause large variability in bee health and therefore induce winter losses^35^. This likely explained why the studied landscape variables did not directly influence final overwintering survival (catch crop: df = 170, z = −1.61, P = 0.91; semi-natural habitats: df = 170, z = 1.53, P = 0.89; Table 1), even if they demonstrably improved the physiological state of bees during the pre-wintering period. Thus, the access to nutritional resources may be important for winter preparation but does not necessarily prevent the detrimental effects of other environmental and sanitary factors before or during the winter (e.g. *Varroa* effects, as shown above and by Dolezal *et al*.^30^).

This study provides a first tentative reconstruction of the ecophysiological basis of overwintering survival. Although the number of different landscape contexts actually covered in our study is currently moderate (18 contexts, including 13 with detailed physiological data), we could recover here the most important correlate of overwintering mortality (*Varroa* mites). We have also shown that the landscape quality is liable to influence the physiological state of honey bees. This candidate model should however be reevaluated with a greater amount of apiaries, enlarged range of biogeographical contexts and foraging distances to confirm that physiological state may be used as a surrogate of future colony survival or collapse risk. This should be coupled with a scale dependency analysis to model and assess the optimal spatial management grain for effective landscape restoration.

### Effects of landscape enrichment on pollen diets

We performed palynological identifications and nutritional evaluation as an *a posteriori* analysis for confirming the use of melliferous catch crop by bees, and as an additional source of information to help understand the observed links between landscape and colony state of health. Landscape enrichment with the melliferous catch crop treatment (Table 2) improved the physiological state of bees during the pre-winter period, likely due to higher pollen diet diversity rather than better nutritional abundance and quality (Table 3). In the absence of pollen resource landscape enhancement, pollen diet was largely dominated by climbing ivy (*Hedera helix*) (71% of pollen diet volume on average, and >90% in half of the samples) (Tables 2 and 3). When available in the foraging range, melliferous catch crop accounted for, on average, 52% of a colony total pollen diet composition, thereby offsetting significantly the broad dominance of climbing ivy pollen (−39% relative change) and increasing significantly pollen species diversity (+87% in species diversity, +77% in species evenness, Table 3). However, the daily pollen intake did not increase significantly and the overall pollen diet energy and protein contents remained unchanged (Table 3). Although, we could not exclude the possibility that the diets provided by the melliferous catch crop treatment were of higher quality regarding other nutrients (e.g. lipids, amino acids, vitamins).

Monofloral and diversified diet, have long been suspected to differentially affect bee health. While some studies revealed, via experimental manipulation of diet composition, that pollen diet diversity increases bee immunocompetence^39^ and reduces disease susceptibility^40^,^45^, it still remains unclear whether the benefits of resource diversity apply at the field level, which is the key for supporting decision-making toward the management of natural resources. Although dedicated experiments will be needed to elucidate this question, our results suggest that pollen diversity might provide benefits to bee health in natural conditions and at the landscape level. Those results were further supported by the even stronger effect of semi-natural habitats, which generally offer a great diversity of floral resources^9^,^46^,^47^.

The underlying mechanisms linking pollen diversity to bee health have yet to be determined. However, De Groot showed that honey bees require a set of essential amino acids in specific proportions for normal growth and development^48^. In addition, bumble bee larvae become heavier when fed with polyfloral pollen diets compared to larvae fed with monofloral diets, even with higher protein content^49^. Therefore, it is possible that an increase in environmental plant species diversity optimizes the occurrence, diversity and/or proportion of specific pollen nutrients (proteins, amino acids, lipids, starchs, sterols, vitamins and minerals) that are required for the development of certain physiological traits, such as fat body and vitellogenin production.

## Conclusions

The ubiquity of habitat degradation requires identifying potential landscape patterns that may act as stressors for bees and providing recommendations for habitat restoration^10^,^50^. Within the scope of this experimental design, we found that bee health is better influenced by semi-natural habitats than by landscape enrichment with catch crops. This suggests that, when considering habitat restoration, artificial bee pastures may be designed as a complementary management measure intended to support semi-natural habitat protection and restoration.

Our study also indicates that applying ecophysiological approaches to honey bee and native bee conservation might be complementary to the more conventional distribution-based studies, which have shown that agri-environmental schemes promoting the conservation of semi-natural habitats or the development of flower-rich field margins in farming areas are favourable to bee abundance and diversity^51^,^52^,^53^,^54^,^55^ (but see ref. 56). Adding a physiological dimension to the environmental variables will benefit the assessment of population health and sustainability^20^,^21^. In conclusion, this work highlights landscape ecophysiology as a promising field of research for better understanding the influence of the environment on pollinator health and setting the stage for more effective pollinator conservation.

## Methods

### Experimental set-up and colony monitoring

We monitored the overwintering survival of 350 honey bee colonies (163 and 187 colonies over winters 2012–13 and 2013–14, respectively) in an intensive farming system from central western France, Centre French region (see supplementary Table S1, Figs S1 and S2). Colonies belonged to three volunteer professional beekeepers from different parts of the region located 80 to 110 km apart. In each beekeeping set-up and each year, a 1.5 km radius area (approximate honey bee foraging range in autumn^57^) was experimentally enriched with 5.0 to 31.5 ha of melliferous catch crops during the pre-wintering period (mid-September to mid-October). Colonies were set up to prepare for winter either inside the melliferous areas (n = 184 colonies split into 10 apiaries) or 8–10 km farther away with exclusively non-melliferous catch crop fields within the foraging range (n = 166 colonies split into 8 apiaries), following a control-*vs*-treatment experimental design with 2 to 4 simultaneous apiary monitoring (supplementary Table S1 and Figs S1 and S2). Details on the species composition of catch crops (*Avena sativa, Brassica juncea, Helianthus annuus, Phacelia tanacetifolia, Sinapis alba, Trifolium alexandrinum, Vicia benghalensis, Vicia sativa*) are shown in the supplementary Table S2. The allocation of colonies among apiaries was random, but all colonies within a given paired (control-*vs.*-treatment) experimental set-up originated from the same professional beekeeper, and therefore had shared the same management history and honey production (rapeseed and acacia) during the season. Colonies were different between years.

The monitoring started during the week preceding the expected onset of catch crop flowering (mid to end of September, depending on the study year). All colonies received a standardized *Varroa* mite treatment (APIVAR^®^, Véto-pharma) and their initial state of brood development was documented by tallying the amount of beehive frame sides (out of 20) covered with brood. Four weeks later, at the end of flowering (mid to end of October), brood area was reassessed and adult honey bees were sampled to determine their physiological state and *Varroa* infestation levels (see methods below). Finally, as cold days arrived, colonies were gathered back into their respective beekeeping domain to standardize overwintering conditions. Overwintering was considered successful for colonies that remained operational for beekeeping activity in early spring, therefore excluding collapsed and weak or orphan colonies. Colony survival was related with physiological state, brood area, *Varroa* infestation and the surrounding landscape characteristics.

### Landscape quality

Beside catch crop land cover, landscape quality was documented by quantifying the extent of permanent semi-natural habitat (woodlots and hedgerows) within the 1.5 km foraging range around the apiaries. Geographical information on semi-natural habitats was obtained from the French national remote sensing database on vegetation layers (Institut Géographique National) and processed with the Quantum GIS mapping software version 2.2.

### Physiological traits and Varroa infestation assessment

To determine the phoretic *Varroa* mite infestation rates of colonies, around 200 bees were collected and washed with soapy water (TEEPOL) to dislodge the mites for counting^58^. Infestation rate was reported as the number of mites per 100 adult bees (n = 339 colonies from 18 apiaries).

Additionally, at the end of the flowering period, around 100 adult bees per colony (n = 175 colonies from 13 apiaries) were collected on brood frames, placed in dry ice and stored at −80 °C. Fat body quantification was performed on a pool of 30 abdomens using the ether extraction method described in^59^. The expression level of vitellogenin was determined by quantitative RT-PCR. For each colony, three pools of 10 abdomens were each homogenized in 1 ml of Qiazol reagent (Qiagen) with a TissueLyser (Qiagen) (4 × 30 s at 30 Hz). The homogenates were incubated for 5 min at room temperature and after centrifugation (12,000 g for 30 s at 4 °C) the 3 supernatants were pooled (167 μl each, giving a 501 μl supernatant). RNA extraction was then carried out as indicated in the RNeasy Plus Universal kit (Qiagen). cDNA synthesis and analysis of vitellogenin expression level was performed as in^40^. Cycle threshold values of vitellogenin were normalized to the geometric mean of the housekeeping genes actin and eIF3-S8 using the comparative quantification method (delta Ct method). We used published sequences of primers for vitellogenin^60^, actin^61^ and *eIF3-S8*^62^.

### Palynological validation and nutritional characteristics

To further ascertain the use of melliferous catch crops by foraging honey bees and to document the possible contribution of pre-wintering nutrition to bee health and overwintering survival, we sampled pollen from one to five (depending on apiary size) randomly chosen colonies per apiary (n = 27 colonies from 10 apiaries). On the second week of catch crop flowering, standard pollen traps were placed at the hive entrances to collect pollen loads during three to six consecutive days (depending on microclimatic conditions), in order to cover three sampling days suitable for foragers (temperature >15 °C, low wind and no rain) and to reach at least 15 g pollen samples. Samples were stored at −20 °C. Two 4-g subsamples per colony were used for the pollen species identification as described in Requier *et al*.^46^. Briefly, pollen samples were diluted in water and mounted onto microscope slides, which were stained with Fuschin, and examined at 400x magnification. Pollen was identified to genus and, when possible, to species. At least 300 pollen grains were counted and identified on each slide. The identification process was duplicated and averaged for improving accuracy. The relative contribution of each pollen species to the total volume of collected pollen in a sample was determined based on pollen grain size^46^. Protein and energy content were determined on 5 g of homogenized pollen subsamples the year of sampling as in Requier *et al*.^46^.

### Statistical analyses

Data were analyzed using generalized linear mixed models (GLMMs) with either a binomial family error distribution for binary data (overwintering success) or a Gaussian family for quantitative data, beforehand log_2_-corrected to recover normality whenever necessary (brood area, vitellogenin level). By specifying a random grouping structure in the dataset, GLMMs allow proper accounting for the non-independency of colonies from the same apiary and the beekeeping set-up.

We then produced a tentative path model linking survival with the studied variables: physiological traits, *Varroa* infestation, brood area and landscape. In particular, we expected that *i*) overwintering survival would increase with vitellogenin level, itself increasing with fat body content and that *ii*) each step of this causal chain would be potentially influenced either negatively by *Varroa* infestations, or positively by landscape quality and initial or final (post-flowering) brood area. We also considered the possible concomitant effects of initial brood area and landscape quality on final brood area before winter. Once computed, the tentative path model was refined by dropping non-significant links and by sequentially adding any link that was initially ignored until the path model was judged statistically supported by the data. New links were added by order of increasing P-value, and deviation from expected conditional independence assessed using the d-separation test^41^ specially suited for generalized mixed models^42^. All quantitative explanatory variables were standardized to a range [0, 1], so that coefficient estimates can be readily compared to determine the most influential explanatory variables in the candidate path models.

We finally performed *a posteriori* comparisons of pollen diet characteristics (pollen intake (g.day^−1^), composition (species richness *S*, Shannon diversity index *H’* and Pielou’s evenness index *J’*) and nutritional properties (energy and protein content)) among catch crop treatments using Kruskal-Wallis tests. All analyses were performed with the R software version 3.1.1^63^.

## Additional Information

**How to cite this article**: Alaux, C. *et al*. A ‘Landscape physiology’ approach for assessing bee health highlights the benefits of floral landscape enrichment and semi-natural habitats. *Sci. Rep.* **7**, 40568; doi: 10.1038/srep40568 (2017).

**Publisher's note:** Springer Nature remains neutral with regard to jurisdictional claims in published maps and institutional affiliations.

## Supplementary Material

Supplementary Information

## Figures and Tables

**Table 1:** Statistical details of selected or missing paths coefficients from the path model analysis.

Response	Predictor	Estimate	SE	DF	Statistics	P
***Selected path coefficients** ( **estimates standardized to data range***)						
Overwintering survival	*Varroa* infestation	−5.552	1.996	170	z = −2.782	0.0054
Overwintering survival	Vitellogenin	3.046	1.265	170	z = 2.408	0.016
*Varroa* infestation	Initial brood area	0.052	0.017	334	t = 3.161	0.0017
Vitellogenin	Melliferous catch crops	0.114	0.033	169	t = 3.486	0.0006
Vitellogenin	Semi-natural habitats	0.178	0.057	169	t = 3.105	0.0021
Vitellogenin	Fat body	0.201	0.068	169	t = 2.944	0.0036
Fat body	Initial brood area	0.162	0.052	169	t = 3.131	0.0021
Fat body	Semi-natural habitats	0.186	0.063	169	t = 2.948	0.0037
Fat body	Melliferous catch crops	0.099	0.035	169	t = 2.836	0.0051
Final brood area	Initial brood area	0.554	0.044	341	t = 12.671	<0.001
Final brood area	Melliferous catch crops	0.109	0.04	341	t = 2.749	0.0063
***Missing path coefficients** ( **estimates standardized to data range***)						
Final brood area	Semi-natural habitats	0.1404	0.0766	340	t = 1.8322	0.0678
*Varroa* infestation	Semi-natural habitats	−0.1227	0.2221	333	t = −0.5526	0.5809
Overwintering survival	Semi-natural habitats	0.7922	0.5186	170	z = 1.5276	0.8994
*Varroa* infestation	Melliferous catch crops	1.7131	1.2636	333	t = 1.3557	0.1761
Overwintering survival	Melliferous catch crops	−3.4473	2.1434	170	z = −1.6083	0.9143
Vitellogenin	Initial brood area	0.1431	0.0966	168	t = 1.4806	0.1406
Overwintering survival	Initial brood area	0.0367	0.2319	170	z = 0.1582	0.3832
Fat body	Final brood area	0.5433	0.552	168	t = 0.9843	0.3263
Vitellogenin	Final brood area	−0.1932	0.1111	167	t = −1.7386	0.0839
*Varroa* infestation	Final brood area	−0.2167	0.1587	330	t = −1.3656	0.173
Overwintering survival	Final brood area	0.6236	0.269	170	z = 2.3183	0.9837
*Varroa* infestation	Fat body	−0.0224	0.0323	167	t = −0.6945	0.4883
Overwintering survival	Fat body	−0.0548	0.0432	170	z = −1.2679	0.838
*Varroa* infestation	Vitellogenin	−0.1077	0.1606	166	t = −0.6709	0.5032

The estimate standardized to data range and corresponding P-value are shown for each path. The path model includes survival, physiological, *Varroa* infestation, brood and landscape variables.

**Table 2:** Average species and morphotype composition of the pollen volume collected by honeybees in melliferous and non-melliferous catch crop treatments.

Pollen species or morphotypes	Melliferous catch crop treatments	Absence of melliferous catch crop treatments
**Pollen species or morphotypes potentially originating from the melliferous catch crops**		
*Helianthus annuus* (Asteraceae)	0.1%	
*Phacelia tanacetifolia* (Hydrophyllaceae)	3.2%	
*Trifolium alexandrinum* (Fabaceae)	16.9%	
type *Sinapis*/*Brassica* sp. (Brassicaceae)	32.0%	2.3%
**Other pollen species**		
*Ammi majus* (Apiaceae)	<0.1%	
*Castanea sativa* (Fagaceae)	<0.1%	<0.1%
*Datura stramonium* (Solanaceae)		<0.1%
*Fagopyrum esculentum* (Polygonaceae)*		<0.1%
*Guizotia abyssinica* (Asteraceae)*	<0.1%	0.1%
*Hedera helix* (Araliaceae)	43.7%	73.3%
*Mercurialis annua* (Euphorbiaceae)		2.0%
*Papaver rhoeas* (Papaveraceae)	<0.1%	
*Plantago lanceolata* (Plantaginaceae)		0.1%
*Raphanus sativus* (Brassicaceae)*	3.7%	18.9%
*Reseda lutea* (Resedaceae)	0.1%	0.1%
type *Bellis perennis* (Asteraceae)		<0.1%
type *Brassica napus* (Brassicaceae)*	0.1%	
type *Calendula* sp. (Asteraceae)		0.1%
type *Cichorium* sp. (Asteraceae)	0.1%	0.6%
type *Crepis* sp. (Asteraceae)		0.1%
type *Pinus* sp. (Pinaceae)	<0.1%	<0.1%
type *Raphanus raphanistrum* (Brassicaceae)	0.6%	1.9%
type *Rhamnus* sp. (Rhamnaceae)	<0.1%	
type *Rubus* sp. (Rosaceae)	0.1%	
type *Solanum* sp. (Solanaceae)*	0.1%	0.6%
type *Trifolium* pratense (Fabaceae)	<0.1%	
type *Veronica* sp. (Scrophulariaceae)		0.1%
type *Viola* sp. (Violaceae)		0.7%

In the “Other pollen species” section, pollen originating from crop plants are indicated by an asterisk; Other pollens are likely originating from semi-natural habitats.

**Table 3:** Comparison of mean (±SD) pollen diet characteristics between catch crop treatments.

Pollen diet properties	MCC treatments	Absence of MCC treatments	Kruskal-Wallis statistics
Daily pollen trap content (g)	23.82 ± 33.13	13.19 ± 16.82	χ^2^ = 0.68, n = 25, P = 0.41
Pollen diet volume (%) from MCC species	51.99 ± 27.91	2.35 ± 5.58	χ^2^ = 16.74, n = 25, P < 0.001
Species richness (*S*)	5.2 ± 2.1	4.4 ± 1.8	χ^2^ = 0.83, n = 25, P = 0.36
Species diversity (Shannon *H’*)	1.18 ± 0.53	0.63 ± 0.48	χ^2^ = 5.47, n = 25, P = 0.019
Species evenness (Pielou *J’*)	0.55 ± 0.24	0.31 ± 0.23	χ^2^ = 4.97, n = 25, P = 0.026
Energy content (kcal/kg of dry matter)	5085.6 ± 266.7	5024.4 ± 161.9	χ^2^ = 0.14, n = 17, P = 0.70
Protein content (% of dry matter)	27.78 ± 2.00	27.85 ± 2.58	χ^2^ = 0.15, n = 17, P = 0.69
*Hedera helix* pollen diet volume (%)	43.33 ± 27.91	71.42 ± 33.87	χ^2^ = 4.05, n = 25, P < 0.044

MCC: Melliferous catch crop; Species richness S stands for the number of species or morphospecies; Species diversity *H’* approaches 0 as differences increase among pollen species (or morphospecies) relative abundances, and gets higher as relative abundances become more equal; Species evenness *J’* standardizes *H’* back to the range [0, 1] for easier interpretation, with a value of 1 meaning perfect equidistribution of abundances among species.
