# Supplementary material for: A ‘Landscape physiology’ approach for assessing bee health highlights the benefits of floral landscape enrichment and semi-natural habitats
Source: Sci Rep. 2017 Jan 13;7:40568. doi: 10.1038/srep40568 (PMC5234012; doi:10.1038/srep40568)
Supplement: Supplementary Information [file srep40568-s1.pdf]

A 'Landscape physiology' approach for assessing bee health highlights the benefits of floral landscape enrichment and semi-natural habitats

Cédric Alaux[1, 2*], Fabrice Allier[2, 3], Axel Decourtye[2, 3, 4], Jean-François Odoux[5], Thierry Tamic[5], Mélanie Chabirand[5], Estelle Delestra[6], Florent Decugis[1], Yves Le Conte[1, 2], Mickaël Henry[1, 2]

[1] INRA, UR406 Abeilles et Environnement, Domaine Saint-Paul, CS 40509, 84914 Avignon, France

[2] UMT PrADE, CS 40509, 84914 Avignon, France

[3] ITSAP-Institut de l'Abeille, Domaine Saint-Paul, CS 40509, 84914 Avignon, France

[4] ACTA, CS 40509, 84914 Avignon, France

[5] INRA, UE1255 Entomologie, 17700 Surgères, France

[6] ADAPIC, Cité de l'Agriculture, 45921 Orléans, France

*Author for correspondence: cedric.alaux@inra.fr

Supplementary Table S1. Detailed experimental set up. Colony sample sizes and data availability for the studied variables, and corresponding landscape characteristics.

			Sample sizes and data availability									Land cover (km^2) variables	
**Apiary**	**Beekeeping set-up**	**Survey winter**	**Surveyed colonies**	**Successful overwintering**	**Brood area data**	**Physiological data**	**Varroa infestation data**	**Mean Varroa infestation rate (/100 honey bees)**	**Pollen mass data**	**Pollen composition data**	**Pollen nutritional value**	**Catch crop**	**Semi-Natural habitats**
1	North-East Orléans	2012-2013	30	25	30	30	30	0.25	5	5	1	0	0.114
2	North-East Orléans	2013-2014	31	30	31	30	31	0.45	4	3	4	0	1.171
3	North-East Orléans	2012-2013	30	25	30	30	30	0.07	3	3	3	0.153	0.542
4	North-East Orléans	2013-2014	16	12	16	14	15	0.67	4	2	2	0.315	1.163
5	North-East Orléans	2013-2014	16	15	16	15	15	0.53	0	1	2	0.315	0.735
6	South Chartres	2012-2013	28	15	25	0	27	1.17	0	0	0	0	0.167
7	South Chartres	2013-2014	16	10	16	8	15	5.28	0	0	0	0	0.167
8	South Chartres	2013-2014	16	13	15	4	14	2.54	0	0	0	0	0.145
9	South Chartres	2012-2013	30	25	30	0	30	0.38	0	0	0	0.05	1.248
10	South Chartres	2013-2014	16	14	16	7	15	4.37	0	0	0	0.097	1.248
11	South Chartres	2013-2014	16	13	16	8	14	4.4	0	0	0	0.097	1.249
12	West Orléans	2012-2013	15	11	15	0	15	0.5	3	3	0	0	0.772
13	West Orléans	2013-2014	15	13	15	7	15	1.34	1	1	1	0	0.772
14	West Orléans	2013-2014	15	14	15	8	14	1.23	1	1	1	0	2.652
15	West Orléans	2012-2013	15	12	15	0	14	0.4	4	3	1	0.232	0.04
16	West Orléans	2012-2013	15	14	15	0	15	0.39	0	1	1	0.232	0.513
17	West Orléans	2013-2014	15	12	15	7	15	1.58	1	1	1	0.258	0.358
18	West Orléans	2013-2014	15	9	15	7	15	2.82	1	1	0	0.258	0.043

Supplementary Table S2. Plant species composition of the melliferous catch crop seed mix. The catch crop seed mix was specially customized by Jouffray-Drillaud© (Cissé, France) to combine efficient nitrogen fixation and bee attractivity. All plant species are visited by bees, except oat (*Avena sativa*) whose function is to allow better soil structuration and nitrogen fixation by the seed mix.

**Species**	**Seed mix density (kg.ha-1)**	**Seed mix composition (%, wg/wg)**
Avena sativa (Poaceae)	6,25	25
Brassica juncea (Brassicaceae)	0,75	3
Helianthus annuus (Asteraceae)	2,5	10
Phacelia tanacetifolia (Hydrophyllaceae)	2	8
Sinapis alba (Brassicaceae)	1	4
Trifolium alexandrinum (Fabaceae)	3	12
Vicia benghalensis (Fabaceae)	5	20
Vicia sativa (Fabaceae)	4,5	18

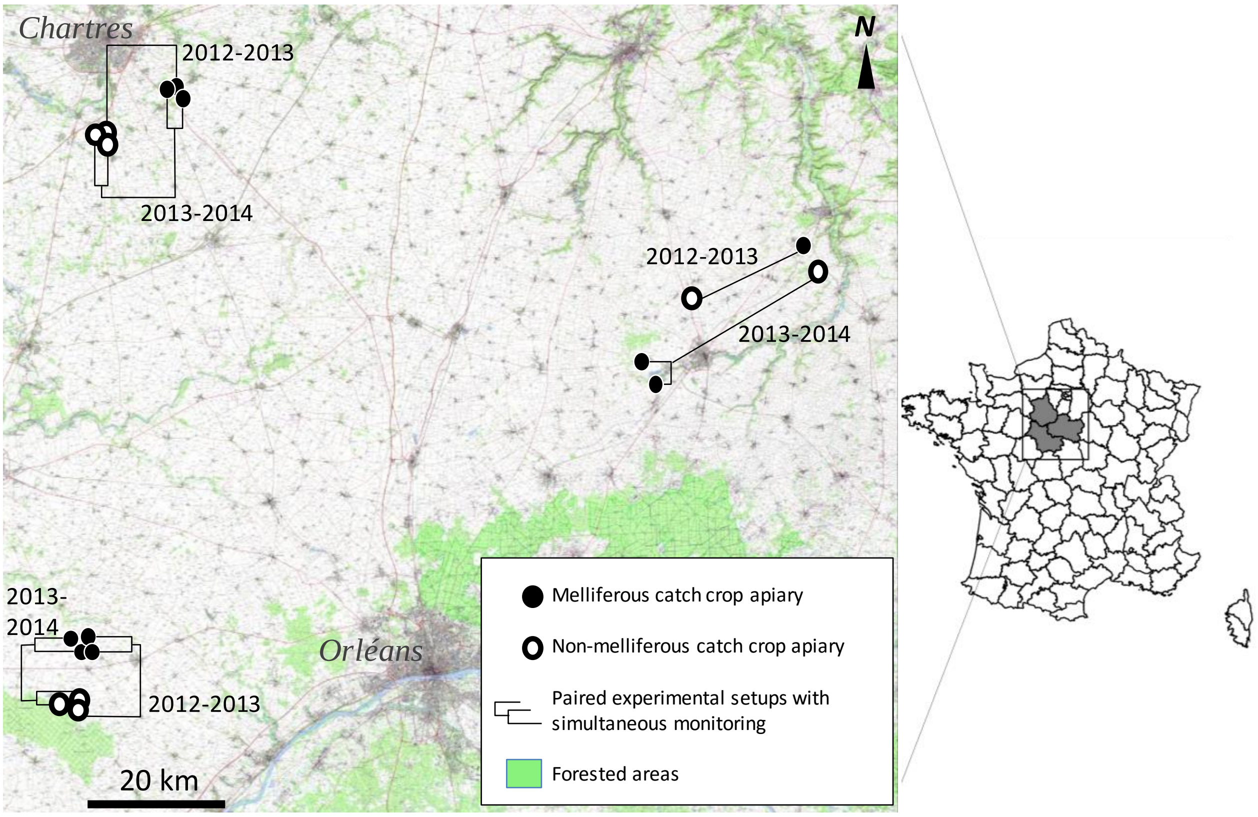

Supplementary Figure S1. Location of the 18 experimental apiaries on a topographic map of the French Centre region. The wintering year is indicated for each paired experimental setup. Geographic data (BD Scan25 IGN 2011/BD Topo IGN 2011) were processed with the Quantum GIS mapping software version 2.2 (http://www.qgis.org/).

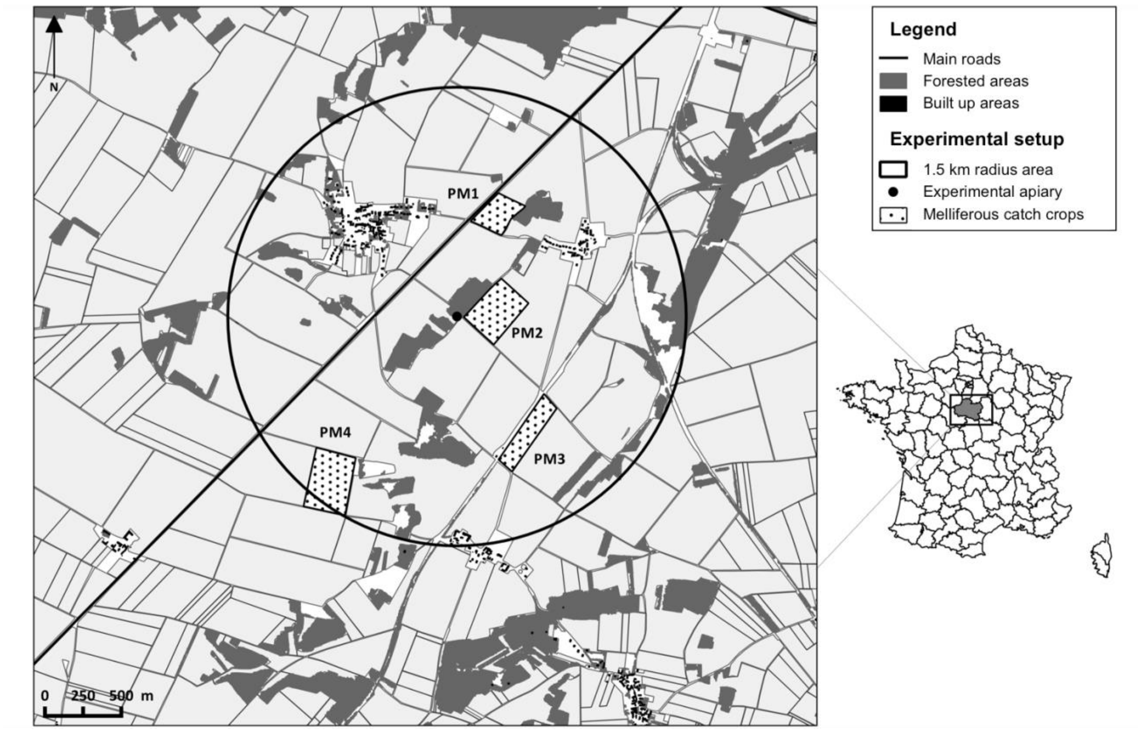

Supplementary Figure S2. Example of an experimental set-up with four melliferous catch crop fields (PM1 to PM4) located within a 1.5km area around a monitored apiary. Geographic data (BD Topo IGN 2011/BD GeoFla IGN 2011) were processed with the Quantum GIS mapping software version 2.2 (http://www.qgis.org/).

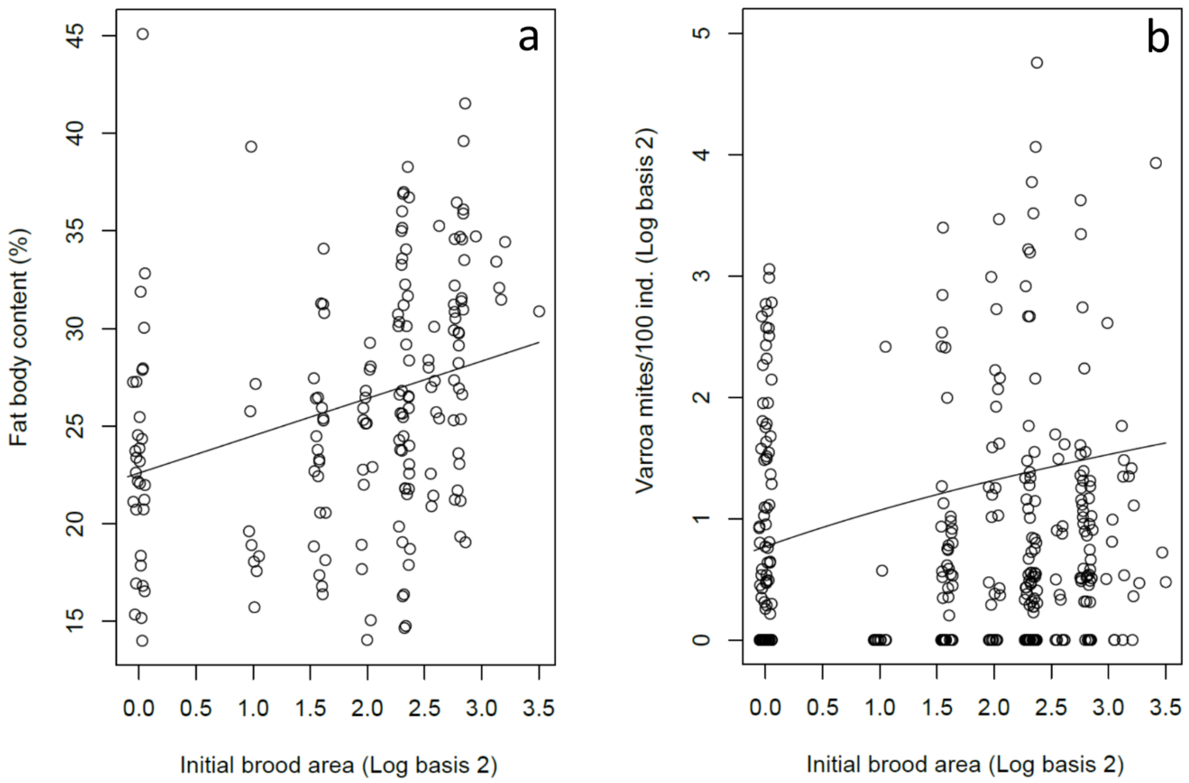

Supplementary Figure S3. Representation of the path model links driven by the initial brood area. Fat body mass (a), and *Varroa* mites infestation level (b) were positively influenced by the colony initial brood area. The continuous lines show model predictions.

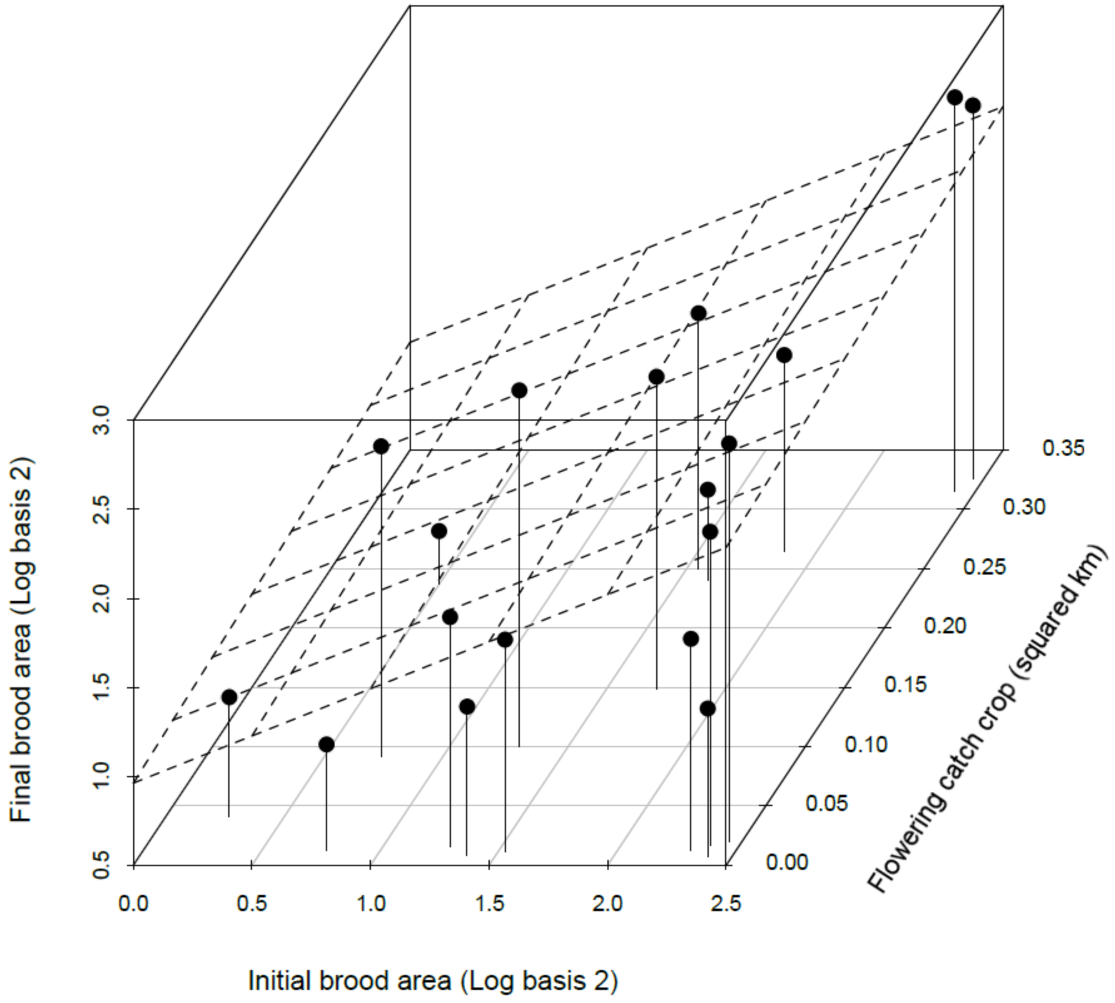

Supplementary Figure S4. Representation of the path model links driving final colony brood area, after the catch crop flowering period. Brood area values (number of frame sides occupied by brood) were averaged per apiary. Trends are depicted by a regression plane. A slight horizontal jitter was applied to separate overlying data.
